# A comparison of erythrocyte sedimentation rates of bloods anticoagulated with trisodium citrate and EDTA among TB presumptive patients at the University of Gondar comprehensive specialized hospital, northwest Ethiopia

**DOI:** 10.1186/s13104-020-04963-0

**Published:** 2020-02-27

**Authors:** Zegeye Getaneh, Fekadu Ayelgn, Geletaw Asemahegn, Habtamu Geleta, Aregawi Yalew, Tadele Melak

**Affiliations:** 1 Department of Hematology and Immunohematology, School of Biomedical and Laboratory Sciences, College of Medicine and Health Sciences, University of Gondar, P.O. Box 196, Gondar, Ethiopia; 2 School of Biomedical and Laboratory Sciences, College of Medicine and Health Sciences, University of Gondar Hospital, P.O. Box 196, Gondar, Ethiopia; 3 Department of Clinical Chemistry, School of Biomedical and Laboratory Sciences, College of Medicine and Health Sciences, University of Gondar, P.O. Box 196, Gondar, Ethiopia

**Keywords:** Erythrocyte sedimentation rate, EDTA, Tri-sodium citrate, Westergren method, Comparison

## Abstract

**Objective:**

The purpose of this study was comparing the erythrocyte sedimentation rate (ESR) results of trisodium citrate (TSC) and ethylene diamine tetra-acetic acid (EDTA) anticoagulants. A comparative cross-sectional study was conducted at the University of Gondar specialized referral hospital, northwest Ethiopia. A total of 70 TB presumptive participants were recruited. From each of the 70 participants of the study, 3 and 1.6 ml of blood was collected in EDTA tubes and 0.4 ml of trisodium Citrate anticoagulant containing test tubes, respectively.

**Results:**

The mean ± SD values of ESR were 57.9 ± 41.45 mm/h in EDTA and 50.99 ± 43.5 mm/h in TSC anticoagulated blood. The mean difference of ESR values between EDTA and TSC blood (6.91 ± 13.66 mm/h) was statistically significant. The Mean ± SD of ESR values using EDTA and TSC in males were 59.57 ± 42.31 and 53.57 ± 44.61 mm/h while for females it was 54.71 ± 40.44 and 46.04 ± 41.82 mm/h, respectively. The study indicated that there was a significant difference between ESR values with EDTA and TSC anticoagulants.

## Introduction

Erythrocyte sedimentation rate (ESR) is a common inexpensive, sensitive and non-specific hematology test frequently ordered in clinical medicine. It is the most widely used laboratory test for evaluating and monitoring the courses of infections, acute phase inflammation, autoimmune and malignant diseases. In addition, it serves as a general sickness index when it is used in conjunction with patient’s clinical history and physical examinations [1–6].

Increased amounts of the plasma proteins, like fibrinogen are the major factors which increase ESR test results by reducing the negative electrostatic force among red blood cells (RBCs), leading to an increased rate of rouleaux formation and the easy falling down of RBCs within plasma [7, 8]. In cases of inflammatory and infectious processes, fibrinogen concentration in the blood increases rouleaux formation and RBCs settle faster than normal [9, 10].

An elevated ESR is often observed in cases of infectious diseases (Tuberculosis (TB), bone and severe skin infections), malignancy, inflammatory or destructive processes, auto immune diseases and rheumatic fever [11–13]. As well as in collagen vascular and infective endocarditic diseases [12, 13]. It is also important for the assessment of the severity of inflammatory bowel diseases in children [14–17] and marking responses to the treatment of TB [18, 19]. Moreover, an increased ESR can be an early predictive marker of HIV seropositive progression towards AIDS [20, 21]. Besides, increased ESR can also be used as an inexpensive “sickness index” in the elderly [22].

A raised result of ESR is observed in a wide range of infectious, inflammatory, generative and malignant conditions and associated occurrences, like anemia, pregnancy, hemoglobinopathies, hemoconcentration and the treatment of anti-inflammatory drugs which change plasma proteins that increase in fibrinogen, immunoglobulin, and C-reactive protein [23, 24].

The principle of the ESR determination is based on the measurements of sedimentation rates of aggregated erythrocytes in plasma. The International Council for Standardization in Hematology (ICSH) recommended the Westergren method as a choice for ESR determination. When anticoagulated blood is placed in a Westergren tube in vertical column, the erythrocytes normally settle quite slowly by the influence of gravity, and the distance of erythrocytes falls down in a vertical column from the plasma in within 1 h will be taken as ESR value [25–27].

For ESR determination, blood anticoagulated with 3.8% trisodium citrate (TSC) or ethylene diamine tetra acetic acid (EDTA) can used. Using undiluted blood anticoagulated with K3EDTA is a recommended specimen for ESR determination by ICSH because it gives a more reliable result than the traditional TSC [21, 28] by reducing the risk of pre-analytic mistakes due to a partially coagulated specimen or small clots, an altered blood/trisodium citrate (TSC) ratio, and problems linked to the final volume, inherent mainly in techniques using special tubes for both specimen collection and ESR measurement [25, 26]. However, according to the 2011 ICSH recommendations, the reference method for the measurement of the ESR should be based on the Westergren method with modifications that use either whole blood anticoagulated with EDTA and later diluted with TSC or saline or whole blood anticoagulated with TSC in a 4:1 dilution ratio in Westergren pipettes [29].

Despite the fact that the ESR test is one of the commonest investigations carried out in the clinical hematology laboratories, there has been no recognized standard control sample for monitoring the test. The reliability and reproducibility of the results depends on the use of correct methodologies. Although, a variety of research was conducted on a matters relating to ESR values of different diseases, no attempts were made to compare the ESR values of blood mixed with TSC and EDTA in the study area. So, this study aimed to assess the variability of ESR values of the two commonly used anticoagulants in individuals suffering from presumptive TB.

## Main text

### Materials and methods

#### Study setting and population

A comparative cross-sectional study was conducted on a total of 70 study subjects who visited the TB Clinic at the University of Gondar comprehensive specialized hospital (UOGCSH) from March to May 2018. UOGCSH is found in Gondar town, Amhara regional state, northwest Ethiopia. Gondar town is located at 727 km from Addis Ababa and 185 km from Bahir Dar town and situated at a latitude and longitude of 12° 36′ N and 37° 28′ E, respectively, with an elevation of 2133 m above sea level [30] to the north western part of Ethiopia.

All patients aged 15 years and above and willing to participate in the study were included. Patients who did not give enough amounts of blood sample and unwilling to take part were excluded.

#### Sample size determination

A total of 70 participants were recruited for this comparative cross-sectional study. The double population mean formula was used to calculate the required sample size using the mean and standard deviation (SD) of TSC and EDTA taken from other studies [31] with 80% power (0.84) and 95% level of significance (1.96).

#### Data collection and ESR determination

The questionnaire and check lists were prepared in the English language and translated to Amharic, the local language, were used to collect socio-demographic and clinical data. Patient blood was collected in K3EDTA tubes (BD Vacutainer^®^ Glass Tubes) and TSC tubes (BD Vacutainer ESR glass tube). Five ml of venous blood was collected from each participant in K3EDTA and TSC tubes. The TSC blood collection tube contained 3.8% of TSC and that of EDTA contained 8.0 mg of EDTA in it. Then 1.6 ml of the whole blood sample was added to the 0.4 ml of 3.8% TSC solution, and 3.0 ml of blood was added to EDTA tube and immediately mixed by inverting the tubes three times. Then ESR values were measured by the Westergren method. For each participant, two tests of ESR determination were performed from each tube within 2 h in accordance with the ICSH recommendation [29]. We filled the Westergren tube to exactly “0” mark and placed the tube in the rack for 1 h, and ESR results were recorded in mm/h. Finally, results obtained from K3EDTA samples were compared to those of TSC samples.

#### Quality assurance

Standard operating procedures (SOPs) and manufacturers’ instructions were strictly followed for all laboratory activities as much as possible. All blood collection test tubes were checked for expiry dates, and laboratory results were recorded on standard report formats using participant identification number. The data of each patient were reviewed for mislabeling and completeness.

#### Data analysis

EpiInfo version 3.5.4 was used for data entry and SPSS version 20 for analysis. In this study, we used the paired t-test to determine significant differences between whole blood anticoagulated using EDTA and TSC for ESR determination. The mean was used since it is the basis for all statistical computations, and SD was obtained to aid the computation of the t-value to measure the average amount of scatter in a distribution. To establish the relationship between the 2 methods, comparison studies were analyzed by simple least squares linear regression and Pearson’s correlation coefficient to obtain the y-intercept, the slope, and the SD of the regression line (Sy/x). The data were compared by the Bland–Altman analysis [32]. Paired sample t-test at 95% confidence level was used to compare the two methods, and in all cases P-values < 0.05 were considered as statistically significant.

### Results

#### Socio-demographic information

A total of 70 TB presumptive patients aged from 15 to 82 years were included in the study. The majority, 46 (65.7%), of the participants were male and lived in rural areas; 21 (30%) were in the age range of 36–50 years (Table 1).

**Table 1:** Socio-demographic information of the study participants (N = 70)

Characteristics	Frequency	Percent (%)
Sex		
Male	46	65.7
Female	24	34.3
Age in years		
15–25	17	24.3
26–35	18	25.7
36–50	21	30
> 50	14	20
Religion		
Muslim	9	12.9
Orthodox	61	87.1
Residence		
Rural	46	65.7
Urban	24	24.3

#### Comparison of ESR values

The Mean ± SD ESR values of patients according to EDTA and TSC anticoagulated blood were 57.9 ± 41.45 and 50.99 ± 43.5 mm/h, respectively. The ESRs value obtained from EDTA blood were higher than TSC blood for all independent variables. The mean ± SD of ESR value in males using EDTA and TSC blood was 59.57 ± 42.31 and 54.71 ± 40.44 mm/h while for females it was 53.57 ± 44.61 and 46.04 ± 41.82 mm/h (Table 2).

**Table 2:** Paired t-test comparing the Mean ± SD ESR values of the study participants using EDTA and TSC Whole Blood

Variables	Category	Mean and SD of ESR in mm/h		Paired differences Mean ± SD	P-value
		EDTA	TSC		
		Mean ± SD	Mean ± SD		
Sex	M (46)	59.57 ± 42.31	53.57 ± 44.61	6.00 ± 14.09	0.006
	F (24)	54.71 ± 40.44	46.04 ± 41.82	8.67 ± 12.92	0.003
Age in year	15–25 (17)	36.53 ± 36.07	28.88 ± 36.26	7.45 ± 9.99	0.006
	26–35 (18)	56.78 ± 43.76	47.11 ± 45.91	9.67 ± 14.82	0.013
	36–50 (21)	62.52 ± 43.10	59.90 ± 45.23	2.62 ± 13.78	0.394
	> 50 (14)	78.36 ± 32.73	69.43 ± 37.09	8.93 ± 15.59	0.052
Residence	Rural (46)	54.76 ± 41.08	47.80 ± 42.55	6.96 ± 14.83	0.003
	Urban (24)	63.92 ± 42.36	57.08 ± 45.61	6.83 ± 11.38	0.007
Previous TB History	Yes (17)	69.71 ± 41.20	65.00 ± 43.86	4.71 ± 16.60	0.260
	No (53)	54.11 ± 41.20	46.49 ± 42.85	7.62 ± 12.68	0.000
TB Status	Positive (7)	88.71 ± 24.40	82.57 ± 39.94	6.14 ± 16.29	0.357
	Negative (63)	54.48 ± 41.65	47.48 ± 42.76	7.00 ± 13.49	0.000
Total		57.90 ± 41.45	50.99 ± 43.52	6.91 ± 13.66	0.000

*SD* standard deviation

The paired sample t-test analysis showed that there was a statistically significant difference between the mean ± SD of ESR values of the two anticoagulated blood. The mean difference of ESR values between the use of EDTA and TSC anticoagulated blood was 6.91 ± 13.66 mm/h with a t-value of 4.24 (P < 0.0001; 95% CI 3.66–10.17). The computed t-value (4.24) was greater than the tabulated or critical t-value (1.667) at 69 degree of freedom and 95% level of significance. Therefore, there was a significant difference between the use of EDTA and TSC as anticoagulants for ESR determination (Additional file 1: Table S1).

**Fig. 1:**
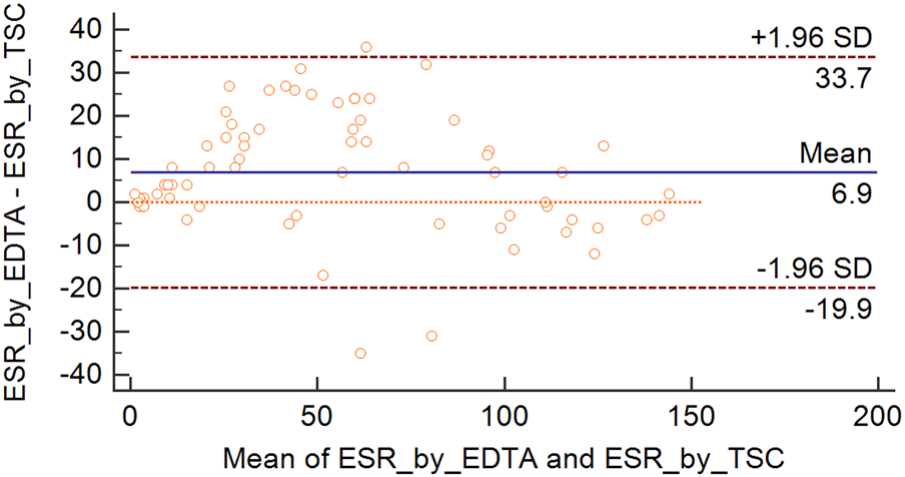
Bland–Altman plot of ESR values from the EDTA and the TSC whole blood using manual Westergren method

The Bland–Altman data analysis showed no systematic bias, and 95% of all samples fell into the narrow 95% limits of agreement (d − 1.96 SD = 6.914–[1.96 × 13.661] = − 19.9 and d + 1.96 SD = 6.914 + [1.96 × 13.661] = + 33.7) (Fig. 1). A linear regression analysis showed a satisfactory correlation between the two methods (r = 0.949, *P *< 0.001; r^2^ = 0.901; y = 11.769 + 0.949x; and Sy/x = 13.106) (Additional file 2: Figure S1).

### Discussion

Although ESR is not a specific marker of inflammation, currently it is frequently used in the diagnosis and evaluation or monitoring of patients with chronic diseases [33]. The ESR value obtained from this comparison showed that the mean value of EDTA anticoagulated blood (57.90 mm/h) was greater than that of TSC blood (50.99 mm/h) by 6.91 mm/h. This may be because EDTA increases rouleaux formation more than TSC, leading to increases in ESR in EDTA blood, or it might be due to a difference in viscosity, where citrated blood may be less viscous than EDTA blood resulting in lower ESR values [34–36]. This observation corresponds to the result reported from Tehran University, Iran, and stated that there was a significant difference between the results of ESR tests which used two anticoagulants [37].

The result of our study was similar to that of a study conducted in Nigeria whose final finding stated that the mean ± SD ESR value of EDTA anticoagulated blood was higher than the mean ± SD ESR value of the TSC. When compared to our finding, the result of the Nigerian study was within the normal range of ESR. The reason for the difference was that their study was conducted on healthy individuals, while ours was conducted on presumed TB patients which increased the ESR value. In addition, the mean ± SD ESR values of males were less than those of females, contradicting the finding. This might be due to the fact that most of our male participants were old and had history of TB [31].

However, the result of our study differed from what was reported in India and stated that ESR measurement values of citrated blood were greater by 4–6 mm/h than those of EDTA [10]. Our result also differed from that of a study conducted at Yenepoya University hospital, India. They found that the values of ESR that utilized EDTA was 4–6 mm/h less than values that used TSC. The authors recommended that ESR be performed using EDTA blood instead of TSC since the former utilizes only a limited amount of blood for the procedure [9]. Another study done in the Philippines found a mean ESR value of 36.7 and 43.09 for EDTA and TSC, respectively. These differed from our findings, perhaps because of some physiological and genetic differences between white and black people [38].

### Conclusion

The average ESR value among participants of UoGCSH using EDTA and TSC blood were 57.90 and 50.99, respectively. The ESR value of EDTA anticoagulated blood was greater than that of TSC blood with a mean difference of 6.91. The computed t-value (4.2.) is greater than the tabulated t-value therefore there is a significant difference between the use of EDTA and TSC as anticoagulant for ESR determination. Therefore, laboratories using EDTA and TSC for ESR determination should have different reference values for each anticoagulant if the observed findings are confirmed in healthy population and among sick individuals.

## Limitations

The study was not only limited to a single district but also dealt with just presumed TB patients. Therefore, the result of this research may not be representative for the whole population.

## Supplementary information

**Additional file 1: Table S1.** Paired Samples t-test of the ESR values of the study participant using EDA and TSC whole blood.

**Additional file 2: Figure S1.** Comparison of the ESR values from the EDTA and TSC whole blood using manual Westergren method by regression analysis (r = 0.949, P = 0.001).

## Data Availability

All the data on which the conclusions of this manuscript are drawn are available in the corresponding author. So that any who needs the data can get it upon reasonable request.

## Abbreviations

CBC: Complete blood count
EDTA: Ethylene diamine tetra acetic acid
ESR: Erythrocyte sedimentation rate
UoGCSH: University of Gondar Comprehensive Specialized Hospital
ICSH: International Council for Standardization in Hematology
K_3_EDTA: Tri potassium ethylene diamine tetra acetic acid
mm/h: millimeter per hour
NCCLS: National committee for clinical laboratory standard
RBC: Red blood cells
SD: Standard deviation
TB: Tuberculosis
TSC: Trisodium citrate
