# Supplementary material for: A comparison of erythrocyte sedimentation rates of bloods anticoagulated with trisodium citrate and EDTA among TB presumptive patients at the University of Gondar comprehensive specialized hospital, northwest Ethiopia
Source: BMC Res Notes. 2020 Feb 27;13:113. doi: 10.1186/s13104-020-04963-0 (PMC7045399; doi:10.1186/s13104-020-04963-0)
Supplement: Supplementary file 1 — Additional file 1: Table S1. Paired Samples t-test of the ESR values of the study participant using EDA and TSC whole blood. [file 13104_2020_4963_MOESM1_ESM.docx]

**Additional file 1: Table S1.**

| **Additional file 1: Table S1.** Paired Samples t-test of the ESR values of the study participant using EDA and TSC whole blood. | | | | | | | | | |
| --- | --- | --- | --- | --- | --- | --- | --- | --- | --- |
|  | | Paired Differences | | | | | t | df | Sig. (2-tailed) |
|  |  | Mean | SD | SE mean | 95% CI the Difference | |  |  |  |
|  |  |  |  |  | Lower | Upper |  |  |  |
| Pair 1 | EDTA - Citrate | 6.914 | 13.661 | 1.633 | 3.657 | 10.172 | 4.235 | 69 | 0.000 |

SD= standard deviation; SE= standard error; t= T-test; df = degree of freedom; 95% CI= 95% confidence interval
