# Supplementary material for: A comparison of erythrocyte sedimentation rates of bloods anticoagulated with trisodium citrate and EDTA among TB presumptive patients at the University of Gondar comprehensive specialized hospital, northwest Ethiopia
Source: BMC Res Notes. 2020 Feb 27;13:113. doi: 10.1186/s13104-020-04963-0 (PMC7045399; doi:10.1186/s13104-020-04963-0)
Supplement: Supplementary file 2 — Additional file 2: Figure S1. Comparison of the ESR values from the EDTA and TSC whole blood using manual Westergren method by regression analysis (r = 0.949, P = 0.001). [file 13104_2020_4963_MOESM2_ESM.docx]

**Additional file 2: Figure S1.**


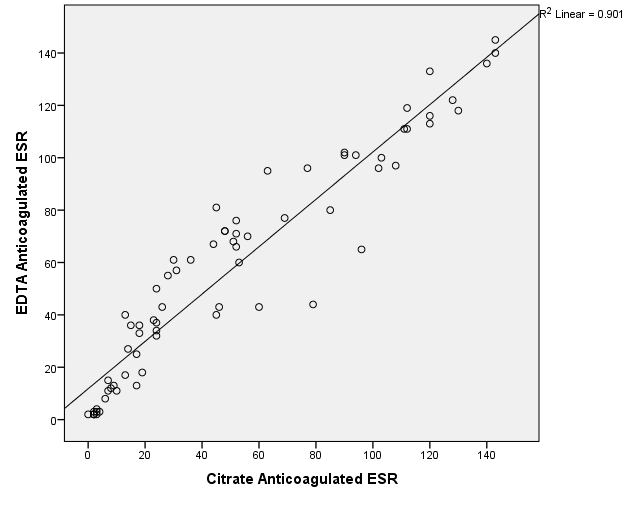


**Additional file 2: Figure S1.** Comparison of the ESR values from the EDTA and TSC whole blood using manual Westergren method by regression analysis (r=0.949, P=0.001).
